# The Heart and Its Examination

**Published:** 1905-01-28

**Authors:** 


### HOSPITAL CLINICS.

### THE HEART AND ITS EXAMINATION.

It has been said that "any fool can hear a murmur," and within the limitations of a dogma the remark is true enough. Zeal in the detection of heart murmurs is far commoner than intelligent effort to interpret them, and Sir William Broadbent did well to dwell upon the high importance of this interpretation in his introductory address to the post graduate audience at the Mount Vernon Hospital. He very wisely insisted on a fact that is obvious enough to be much overlooked, that diagnosis is not an end, but a means; that a patient has no academic interest in the correct identification of a lesion that troubles him, and that his paramount interest is to recover his health, or, failing that, to learn how far his disabilities will interfere with his vital efficiency. There is no need to labour the difficulties of correct interpretation, yet, as the lecturer showed, it is to a great extent not founded on anything more mysterious than rational inference from careful observation. Careful observation is the first essential, and Sir William follows many good teachers in relegating the stethoscope to a place far below the use of the eye and the hands—that is to say, inspection, palpation, and percussion. The following salient points of his address are well worth digestion.

The signs and symptoms of heart affection are in general mutually inter-dependent for their importance, and it is unwise to dissociate them. For instance, a murmur of mitral incompetence may mean much or almost nothing; much, if attended by symptoms such as dyspnœa, almost nothing in a patient who can sustain a sharp burst of physical exertion without undue breathlessness.

The four main symptoms of heart lesions are pain, dyspnœa, palpitation, and dropsy, and of these pain and palpitation are quite useless as indications if considered alone. Pain, for instance, may be anginal or not. Anginal pain is essentially a pain of exertion or emotion, and heart-pain occurring when body and mind are at ease is commonly a reflected symptom of gastric disturbance, particularly of gaseous distension of the stomach; hence the importance of defining the upward limit of the stomach resonance in such cases. If the pain be true angina, the fewer the physical signs found to explain it, the more ominous is the outlook, for the presumption is that sclerosis of the coronary arteries is interfering with the nutrition of the heart.

Palpitation, again, is as often a gastric as a cardiac symptom, as are irregularity and intermission; these can only be interpreted in the light of physical signs, or the state of the arteries as revealed in the pulse.

Dyspnœa, unless otherwise accountable, is in itself evidence of heart disease, even in the absence of signs.

Dropsy is pre-eminently a symptom of mitral incompetence; when it occurs in aortic disease it depends upon secondary dilatation of the mitral ring, and though the mitral valve may be free from structural change it is incompetent, and the lesion is in effect a double one; therefore dropsy in aortic disease is graver than in uncomplicated mitral lesions. Heart murmurs should always be investigated in the recumbent as well as in the upright position of the patient. They may or may not indicate organic disease, and the common insignificant ones are as follows:—

1. A murmur heard during inspiration only.
2. A short, sharp systolic rub heard above and within the apex-beat. This sound indicates a milk-spot on the pericardium.
3. A systolic murmur at the pulmonary base. This sound may be very loud and harsh, but has no significance however loud it be, provided that it is eliminated by a deep inspiration.
4. A systolic murmur at the apex may be due to loss of tone in the heart resulting in relative incompetence of the mitral ring. In these cases the murmur is headed by a good first sound, and so long as this is so the outlook is favourable as regards cure.

True organic murmurs are to be interpreted in the light of their duration, and with reference to symptoms and the other heart sounds. In mitral regurgitation it is usual to find accentuation of the pulmonary second sound owing to congestion in the pulmonary circuit. The greater the accentuation, the worse the lesion.

Again, if the first sound is accompanied by a murmur the lesion is less than when a murmur entirely replaces the sound; for the appearance of the sound shows that the mitral valves are in part, at least, effective. Thus the most favourable mitral lesion is shown by a good first sound followed by a murmur, but unaccompanied by symptoms, or by accentuation of the pulmonary second sound. In such a case the jeopardy to life is minimal.

*Mutatis mutandis*, this argument holds for cases of aortic regurgitation; that is to say, the presence of the second sound is a favourable sign, as is a long-drawn murmur; for both of these indicate that the channel of reflux is small, and the valves therefore partly effective.

